# Does asymmetric gene flow among matrilines maintain the evolutionary potential of the European eel?

**DOI:** 10.1002/ece3.2098

**Published:** 2016-06-30

**Authors:** Miguel Baltazar‐Soares, Christophe Eizaguirre

**Affiliations:** ^1^ GEOMAR Helmholtz Centre for Ocean Research KielDüsternbrooker Weg 2024105KielGermany; ^2^ School of Biological and Chemical SciencesQueen Mary University of LondonMile End RoadLondonE1 4NSUK

**Keywords:** *Anguilla anguilla*, demes, habitat fragmentation, matrilines, population dynamics

## Abstract

Using evolutionary theory to predict the dynamics of populations is one of the aims of evolutionary conservation. In endangered species, with geographic range extending over continuous areas, the predictive capacity of evolutionary‐based conservation measures greatly depends on the accurate identification of reproductive units. The endangered European eel (*Anguilla anguilla*) is a highly migratory fish species with declining population due to a steep recruitment collapse in the beginning of the 1980s. Despite punctual observations of genetic structure, the population is viewed as a single panmictic reproductive unit. To understand the possible origin of the detected structure in this species, we used a combination of mitochondrial and nuclear loci to indirectly evaluate the possible existence of cryptic demes. For that, 403 glass eels from three successive cohorts arriving at a single location were screened for phenotypic and genetic diversity, while controlling for possible geographic variation. Over the 3 years of sampling, we consistently identified three major matrilines which we hypothesized to represent demes. Interestingly, not only we found that population genetic models support the existence of those matriline‐driven demes over a completely panmictic mode of reproduction, but also we found evidence for asymmetric gene flow amongst those demes. We uphold the suggestion that the detection of demes related to those matrilines reflect a fragmented spawning ground, a conceptually plausible consequence of the low abundance that the European eel has been experiencing for three decades. Furthermore, we suggest that this cryptic organization may contribute to the maintenance of the adaptive potential of the species.

## Introduction

The field of evolutionary conservation aims at identifying processes and mechanisms that contribute to the maintenance of the adaptive potential of species and uses this knowledge to improve management (Frankham 2005; Harrisson et al. 2014). The adaptive potential is generally defined as the ability of species/populations to respond to natural selection, whether it relates to species interactions – such as predator**–**preys or host**–**parasite coevolution – or to environmental shifts (Eizaguirre and Baltazar‐Soares 2014) The adaptive potential can be estimated by screening the distribution of genetic diversity across populations of a given species but, for that, some knowledge of those populations' boundaries is needed. Identifying boundaries in natural populations is an extremely challenging but critical task (Waples and Gaggiotti 2006). This is particularly evident in those species with no spatially explicit breeding groups (Manel et al. 2007). For example, reduced gene flow (which implies population structure) may facilitate the evolution of local adaptation (Eizaguirre et al. 2012) but may also hinder the spread of beneficial mutations across a species range, and in extreme cases, may lead to inbreeding depression (Frankham 2005). In contrast, extensive gene flow amongst populations might promote migration load and disrupt patterns of local adaptation (Bolnick and Nosil 2007). Practically, management programs that underestimate the genetic structure of natural populations may prove ineffective and may irreversibly damage the species they aimed to protect (Hutchinson 2008).

Several evolutionary processes are known to establish or reinforce cryptic genetic structures in species with otherwise continuous spatial distributions. Selection (Schluter 2009), mate choice (Kirkpatrick 2000), habitat choice (Via 1999) or sex‐biased dispersal (Pusey 1987; Portnoy et al. 2015) are examples of well‐documented cases. Amongst those, sex‐biased dispersal – which describes situations where one sex shows natal philopatry while the other shows less accurate fidelity to the natal site – is perhaps the best documented (Prugnolle and De Meeûs 2002). Evolutionary lineages of distinct mitochondrial haplotypes (matrilines) are often associated with specific colonies in bats (Rossiter et al. 2005), linked to different nursery areas in sharks (Keeney et al. 2005) or correspond to different nesting groups in turtles (Bowen et al. 2004; Stiebens et al. 2013). While matrilines may differ from each other by as few as one mutation step (Keeney et al. 2005; Levin and Parker 2012), larger differences have also been reported (Tillett et al. 2012). The usual signature of sex‐biased dispersal shows that uni‐parentally inherited loci (such as mitochondrial DNA of female vertebrates) have a stronger signature of differentiation amongst philopatric locations than bi‐parentally inherited loci (Pardini et al. 2001; Bowen et al. 2004).

Theoretically, sex‐biased dispersal is interpreted as an evolutionary mechanism of inbreeding avoidance, whether through the existence of genetic differences between the dispersive and the philopatric sex (Berg et al. 1998) or through the movement of the dispersing sex in order to avoid kin mating (Dobson 2013). Recent evidence showed that despite female philopatry amongst endangered loggerhead turtles, male‐biased opportunistic mating is critical to maintain the genetic diversity – and thus the adaptive potential – of the species by increasing gene flow amongst nesting locations and maintain high genetic diversity (Stiebens et al. 2013). The identification of cryptic genetic structure is therefore essential to estimate the adaptive potential of species.

The European eel (*Anguilla anguilla*) is a highly migratory fish with a life cycle that uses the entire North Atlantic basin. Born in the Sargasso Sea, eels are passively transported towards the European coasts with the main ocean currents. This connection is facilitated by the North Atlantic gyre (Blanke et al. 2012) and, in particular, by an oceanic pathway linking the spawning grounds with the Gulf Stream (Baltazar‐Soares et al. 2014). Upon sexual maturity, all adult eels aim to return to the Sargasso Sea to mate. Recent investigations on speciation and historical demography further reinforce the intrinsic role of the Gulf Stream on this species' evolution (Jacobsen et al. 2014a): by the beginning of the 1980s, the juveniles arriving at European coasts – hereafter referred to as recruitment – experienced a steep decline (Moriarty 1990). This was followed by consecutive years of extremely low recruitment affecting the abundance of adult eels in their continental range (Astrom and Dekker 2007). It is thought that a multitude of factors have contributed to this decline: changes in ocean currents (Baltazar‐Soares et al. 2014) and ocean productivity (Friedland et al. 2007), diseases (Van Nieuwstadt et al. 2001; Kirk 2003), pollution (Robinet and Feunteun 2002), reduced freshwater habitats (Prigge et al. 2013), overfishing (Dekker 2003) and lack of spawners (Dekker 2003) are amongst the most consensual hypotheses.

The European eel population is perceived as a single panmictic reproductive unit (Als et al. 2011) with single‐generation local selection sorting genotypes in European freshwater systems (Pujolar et al. 2014b). Still, after the recruitment collapse, punctual observations of genetic structure amongst coastal locations were detected (Avise et al. 1986; Wirth and Bernatchez 2001; Dannewitz et al. 2005; Baltazar‐Soares et al. 2014). Particularly, the identification of genetically distinct glass eel cohorts by (Dannewitz et al. 2005) was suggested to reflect temporal genetic discontinuities amongst reproductive events. However, the presence of a spatial genetic structure at Sargasso Sea could not be entirely excluded (Dannewitz et al. 2005). This scenario was further extended by the use of hypotheses‐driven ocean models. These models tested predictions of female philopatry to specific locations within the Sargasso Sea as a possible source of genetic structure, by comparing simulated and field data of *A. anguilla* mitochondrial DNA (Baltazar‐Soares et al. 2014). While overall results suggested that the Sargasso Sea may not represent an entirely homogenous spawning ground, field data specifically identified three distinct matrilines. Noteworthy, the definition of a “natal environment” does not need to relate to a given location in the Sargasso Sea, but could rather be associated with imprinting on physical, chemical, or ecological conditions in the region (Miller et al. 2015). Despite successful locations and capture of eel larvae in the Sargasso Sea (Miller et al. 2015), expeditions have been unable either to detect events of reproduction, or capture mature individuals. Realistically, these challenges limit our knowledge regarding the reproductive strategy of this species which thus requires relying on more hypotheses‐driven indirect approaches.

The primary objective of this study is therefore to test for the possibility of the three matrilines identified in (Baltazar‐Soares et al. 2014) to represent cryptic female‐driven demes in the Sargasso Sea. We collected, at a single location in Europe, a total of 403 freshly‐arrived juvenile eels (glass eels) in three consecutive years. Sampling within one location over time has the advantage of controlling for confounding factors associated with varying single generation selection across European coasts (Pujolar et al. 2014b). We then sequenced the same portion of mitochondrial genome as in (Baltazar‐Soares et al. 2014), and screened for the existence of the same matrilines over time. We then grouped individuals per matrilines, measured their relative fitness and used 22 microsatellites to measure genetic differentiation, rates and directionality of gene flow and perform heterozygosity fitness correlations amongst matrilines. We hypothesized that a panmictic population would show bi‐directional gene flow amongst matrilines. Any deviation from this pattern would suggest a different mode of functioning of the eel spawning ground.

## Material and Methods

### Indicator of individual fitness: “condition index after arrival”

We sampled 403 glass eels from three distinct cohorts captured in the mouth of the river Adour in France (GPS coordinates: 43°31′48″N. 1°31′28″W). Individuals were captured within the fourth week of December of 2010 (*n *=* *157), 2011 (*n *=* *127) and 2012 (*n *=* *121). Specimens were dried with absorbing tissue, weighed (total weight ± 1 mg), measured with an electronic caliper (total length, ±0.01 mm), and a piece of tissue was clipped for DNA extraction.

We first calculated the relative condition index (Kn) (Le Cren 1951; Froese 2006) following: Kn* *= *W*/*aL* ^*b*^, where *W* and *L* are weight and length, respectively, *a* is the intercept of the log(*L*) – log(*W*) regression, and *b* is the slope of this regression. To investigate whether we could fit a regression line common to all the cohorts (and therefore consider a species‐specific growth), we first performed an analyses of covariance with log(*W*) as the dependent variable and log(*L*) as an independent variable and “cohort” as a fixed effect. Since all our fish were captured at the glass eel stage in the mouth of the Adour River, we expected Kn to provide an estimate of individual condition after the transatlantic migration. This estimate is referred to as “condition index at arrival”.

### Genetic diversity, structure, and matrilines

All samples were sequenced for the forward and the reverse side of the mitochondrial NADH dehydrogenase 5 (*ND5*) gene following (Baltazar‐Soares et al. 2014). Chromatographs were checked and sequences were assembled and aligned in Codon Code Aligner (v.3.7.1, Codon Code Corporation, Centerville, MA, USA). A total of 355 base pairs of 100% quality (verified by sequencing both directions) was obtained and used for further analyses. In addition, five sequences of American eels (*Anguilla rostrata*), the sister species of the European eel, were added from a previous study (Baltazar‐Soares et al. 2014). Haplotype diversity (Hd) and nucleotide diversity (*π*) were calculated for each cohort in DnaSP v5 (Librado and Rozas 2009). To infer genetic structure, a test of pairwise comparisons of haplotype frequencies amongst cohorts was performed using Arlequin v3.5 (10,000 permutations) (Excoffier and Lischer 2009).

We then compared a panmictic mode of evolution with an alternative scenario. To this end, we used mtDNA matrilines as a proxy signature for philopatric spawning groups, as it is observed amongst animals that follow this strategy. We followed (Baltazar‐Soares et al. 2014) that suggested mtDNA matrilines to reflect female philopatry within the Sargasso Sea. We thus created a mtDNA haplotype list in DnaSP v5 and constructed a network using NETWORK v4.6.1.2 (Bandelt et al. 1999). Five sequences of American eels (*A. rostrata*), the sister species of the European eel were used to provide a visual calibration of intraspecific diversity (Baltazar‐Soares et al. 2014). We calculated a median joining network with the following parameters: frequency criteria inactive, epsilon of 35, and transversions weighted eight times more heavily than transitions, as suggested by analyses of transition/transversions bias performed in Mega v5 (Tamura et al. 2011). Lastly, the network was subjected to a maximum parsimony optimal post‐processing (Polzin and Daneshmand 2003). Connection ambiguities, common in complex and large data sets such as ours (Bandelt et al. 1999), were solved by parsimonious choice of the most frequent connections observed amongst all shortest trees produced by the optimal post‐processing step. Individuals were then grouped into three matrilines (A, B, and C) with respect to their network location and connection to the three most frequent haplotypes. That grouping was also performed within each cohort, which resulted in glass eels to be distributed amongst nine groups (three main matrilines × three cohorts). Hereafter, we will refer to those nine groups as nine “demes”.

### Construction of Bayesian skylines for each matriline

The use of Bayesian statistics (Drummond et al. 2005) and coalescent theory (Kingman 1982) allowed the reconstruction of each matriline's demographic pattern. Demographic history of each matriline was investigated through Bayesian skyline plots (BSP) in BEAST v.1.8 (Drummond and Rambaut 2007). We first estimated the required parameters to run the Bayesian analyses, that is the substitution model, mutation rate and clock model. The substitution model was estimated in jModelTest on the entire dataset (*N *=* *403) (Darriba et al. 2012). The intraspecific mutation rate and clock model were estimated using five *A. rostrata* sequences as an outgroup. For this purpose, we used the tree prior “calibrated Yule mode” (Gernhard 2008) as suggested by the authors when considering sequences from different species (Heled and Drummond 2012). We enforced monophyly of all *A. anguilla* to constrain the tree topology during the course of the Markov Chain sampling (Heled and Drummond 2012). The initial tree root height was set at 5.8 × 10^6 ^years (standard deviation = ±0.5 million years), in line with estimates of most recent common ancestor (TMRCA) of both species reported by (Minegishi et al. 2005) in the most comprehensive phylogenetic analyses of the genus *Anguilla* (Minegishi et al. 2005).

With the substitution model, mutation rate and respective clock model, as well as the initial tree height, we proceeded with the construction of the BSP. BSP were constructed independently for each matriline. The tree prior for these analyses was set to “coalescent: Bayesian skyline” (Drummond et al. 2005). Markov chains were set to 1 × 10^8^ in all the above mentioned analyses. Convergence, that is effective sample size (ESS) >200 and distribution of posterior's marginal likelihood was inspected in Tracer v1.6 (Rambaut et al. 2014).

### Genetic footprints of gene flow amongst hypothetical philopatric demes: insights from nuclear DNA

#### Validation of microsatellite data: null alleles, linkage, and selective neutrality

In addition to the *ND5*, all samples were screened for variation at 22 microsatellites. The amplification was performed in four PCR multiplexes of four to six microsatellite loci developed for the European eel (Wielgoss et al. 2008; Pujolar et al. 2009b; Als et al. 2011) following the protocol described in Text S1. Reactions were performed in a total volume of 10 μL and followed the QIAGEN^©^ Multiplex (Hilden, Germany) PCR kit's recommendations. Genotyping was performed on an ABI^©^ 3100 Genetic Analyzer. Alleles were called in GENEMARKER^©^ v. 1.91 (Softgenetics LLC, State College, PA). We controlled the quality of microsatellite data by estimating the frequency of null alleles through maximum likelihood estimates of gene frequency [Dempster's EM method (Dempster et al. 1977)] and inferring linkage of alleles between pairs of loci. Those analyses were done in GENEPOP (Rousset 2008) and GENETIX (Belkir et al. 1999) respectively.

To validate the use of the 22 nuclear markers for population genetic purposes, we inferred their selective neutrality by exploring the distribution of the *F*_ST_/expected heterozygosity ratio expected to occur in an island model and assuming neutral evolution (Antao et al. 2008). These calculations were performed in LOSITAN (Beaumont and Nichols 1996; Antao et al. 2008). The confidence intervals were calculated with 100,000 permutations and after forced calculation of a mean *F*_ST_, as suggested to improve robustness (Antao et al. 2008). Simulations were performed assuming both an infinite allele and a stepwise mutation model. We considered pairwise comparisons between matrilines and pairwise comparisons between demes, to infer if hypothetically non‐neutral evolving loci would be linked to (1) mtDNA variation and (2) mtDNA variation within cohorts respectively. Lastly, to test for correlation between mitochondrial and nuclear loci, we performed a mantel test with the *F*_ST_ values obtained with both type of markers using the “ape” function in R.

#### Genetic diversity, contemporary demography, and structure

To obtain estimates of genetic diversity, we calculated the observed heterozygosity (He), inbreeding coefficient (*F*_IS_), and rarefied allelic richness (Ar) in GENETIX (1000 bootstrap), (Belkir et al. 1999) and HP‐RARE v1.0 (Kalinowski 2005), respectively, for each deme within a cohort. We also investigated if the recent decline in population abundance had left a genetic signature of decreased diversity in the different demes. For that, we used BOTTLENECK (Cornuet and Luikart 1996), which relies on methods sensitive to recent and severe reduction of effective population size (*N*_e_) (Cornuet and Luikart 1996). A two‐phase mutation model was assumed with 10% of the loci allowed to evolve through stepwise mutations with variance of 10% (Kimura and Ohta 1978).

To evaluate and compare the magnitude of genetic differentiation between nuclear and mitochondrial markers, we calculated the *F*_ST_ amongst cohorts in Arlequin v3.5 (10,000 permutations). We also investigated the distribution of molecular variance amongst the possible nine demes (three cohorts and three matrilines): to this end we performed an AMOVA with “cohort” as higher hierarchical group (Arlequin, 10,000 permutations). Then, we used STRUCTURE v2.3.3. to infer the likelihood of population structure, that is incomplete admixture, without a‐priori knowledge of cohort or matriline (Pritchard et al. 2000). STRUCTURE's running period consisted of 100,000 MCMC after a burn‐period of 10,000 chains. We used an admixture model where *α* (the degree of admixture) was inferred from a uniform prior, with initial *α*= 1, max = 10.0 and standard deviation = 0.025. The frequency model was set to correlated allele frequencies amongst populations. The number of subpopulations (*K*) was set to 9. *K *=* *9 is the maximum number of demes that can be expected from a spatial and temporal combination of three matrilines and three cohorts. Five iterations were run for each *K*. The most likely number of subpopulations present in the dataset was assessed through two methods: (1) calculation of Evanno's *ΔK* (*ΔK* = mean(|*L*″(*K*)|)/SD(*L*(*K*)) (Evanno et al. 2005) in STRUCTURE HARVESTER (Earl 2012) and (2) visual inspection of admixture plot produced by STRUCTURE.

#### Estimates of directionality and migration rates amongst demes

One of the primary goals of this study was to investigate gene flow amongst putative female philopatric reproductive units represented by different matrilines. By grouping the samples by female matrilines, gene flow measured with nuclear markers would primarily be the product of male migration. Here, using Bayesian inference methods implemented in MIGRATE‐n v.3.6.4 (Bertorelle et al. 2009), we compared the likelihood of a panmictic scenario against the hypothetical female‐driven structure. For this, we created two population models. In the first model (model I), the three matrilines were considered to be part of a single panmictic population, while in model II – island model – each matriline was regarded as segregated reproductive unit allowing for bi‐directional gene flow, that is possibility of emigration and immigration to exist. These scenarios were simulated independently for each cohort.

Three replicates were performed for each scenario, and the most likely model was identified by comparing the average marginal likelihoods of each model (Mlog) (Beerli and Palczewski 2010). The effective number of migrants (*N*_em_) was calculated following *N*_em_ = (*Μ*_*j*_ *→*_*i*_ *θ_I_)/4. The priors of the parameters *θ* and *Μ* were the same for both models which allowed the direct comparison of marginal likelihoods. We used an uniform prior of *θ* ranging between 0 and 200, with mean = 200 and delta = 20, after improving over other posterior probability distributions. The uniform prior for *Μ* remained as default, that is ranged between 0 (minimum) and 1000 (maximum), with delta = 100. The running parameters included a long chain of 5000 recorded steps, with an increment every step of 100 over three identical replicates. A total of 1,500,000 parameters were visited. This methodology allows for the calculation of the harmonic mean of marginal log likelihoods (Raftery 1996).

#### Individual‐based genetic indices and relationship with migration rates

At the individual level, we calculated the internal relatedness (IR) and the homozygosity by *loci* (HL) indices in R version 2.13.2 (Fox 2005) using the *Rhh* package (Alho et al. 2010). IR compares parental half genotypes within an individual. It ranges from −1 (outbred) to 1 (inbred), where 0 is the score of individuals born from the random pairing of unrelated parents (Amos et al. 2001). HL is a homozygosity index that extends IR by considering the contribution of each locus, rather than each allele, while estimating allele frequencies (Aparicio et al. 2006). Such difference between both indices might be particularly informative in the presence of migration amongst reproductive units (Aparicio et al. 2006). For instance, since IR weights the contribution of alleles based on their frequency, homozygous individuals carrying rare alleles (brought in the population through migration) are attributed higher IR index than those homozygous individuals carrying more common alleles (Aparicio et al. 2006). Those differences stand out when comparing the strengths of correlation of IR and HL with a population‐based inbreeding coefficient, such as the *F*_IS_. Under asymmetric migration, this would translate in a lower correlation coefficient (*r*) between mean IR and *F*_IS_ in comparison with the correlation coefficient obtained for HL and *F*_IS_. Such a result would suggest that rare or low frequency alleles are transported between demes through migration. Hereafter we will refer to the correlation coefficients *r*(mean IR, *F*_IS_) and *r*(mean HL, *F*_IS_) as *R*_IR_ and *R*_HL_ respectively. Lastly, IR and HL were calculated independently for each deme, ensuring that those metrics were weighed by the allelic frequencies of the deme alone and not of the whole data set.

#### Heterozygote‐fitness correlations

To verify if individual fitness could be explained by matriline or cohort, relationships between individual condition index at arrival (Kn) – measure of relative fitness – and individual genetic indices (HL and IR) were analyzed using linear models, with “matrilines” nested into “cohort”. To avoid confounding effects of HL and IR on the linear model, we calculated the residuals of their correlation and used those residuals as independent variable in the linear model. All statistical analyzes were performed in R 2.13.2 (Fox 2005).

## Results

### Variation in length (*L*), weight (*W*) and condition index (Kn) amongst cohorts

**Figure 1:**
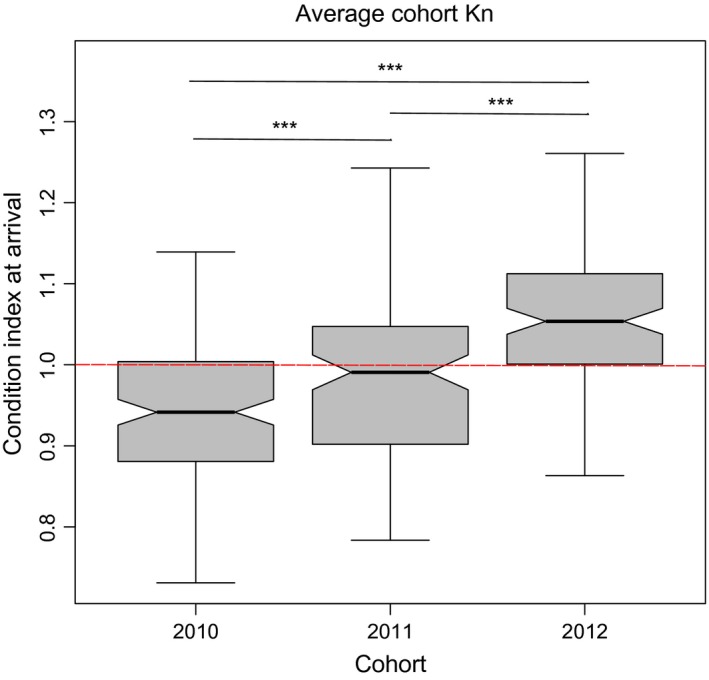
Variation of condition index (Kn) amongst glass eel cohorts. The condition index at arrival (Kn) was calculated with the slope and intercept of the log(*W*)/log(*L*) regression equation built upon the allometric growth of all specimens; ***represents pairwise relationships with *P *<* *0.001. The line at 1.0 represents the mean condition index, following (Le Cren 1951).

Although sampled within the same week and the same location (River Adour, France) every year for three consecutive years, the mean fish length upon arrival at the European coasts significantly varied (ANOVA_cohorts_, *F*_2,401_ = 85.83, *P *<* *0.001, *L*_2010_ = 66.59 ± 3.49 mm, *L*_2012_ = 71.60 ±3.55). Mean fish weight also significantly varied and ranged from 0.23 ± 0.04 g in the 2010 cohort to 0.33 ± 0.05 g in the 2012 cohort (ANOVA_cohorts_, *F*_2,401_ = 184.50, *P* <* *0.001). The analysis of co‐variance supported the use of a single regression line to calculate the condition index (Kn) of all individuals (Table 1 and 2). The comparisons of the condition index among cohorts revealed statistically significant differences in Kn, with higher Kn in the most recent cohort (Fig. 1, ANOVA_cohorts_, *F*_2_ = 48.22, *P *<* *0.001; Post‐hoc tests: *t*_2010–2011_ = 4.065; *t*_2010–2012_ = 9.817; *t*_2011–2012_ = 5.320, all *P *<* *0.001).

**Table 1:** Regression equations fitted for the log(*L*) – log(*W*) relationship in each cohort

Cohort	Equation	*R*	*P*
2010	*y* = 2.817*x* − 2.782	0.73	<0.001
2011	*y* = 2.877*x* − 2.864	0.73	<0.001
2012	*y* = 3.003*x* − 3.055	0.75	<0.001
All	*y* = 3.397*x* − 3.813	0.80	<0.001

**Table 2:** ANCOVA testing for differences in the slope of log(*L*) – log(*W*) relationship amongst cohorts

log(*W*)^~^	*t*‐Value	*P*
log(*L*)	19.724	<0.001
Cohort 2011	−0.250	0.803
Cohort 2012	−0.758	0.449
Log(*L*):cohort 2011	0.328	0.743
Log(*L*):cohort 2012	0.934	0.351

### Evolution and demographic history of the matrilineages

#### Genetic diversity, differentiation and haplotype network

Amongst cohorts, the haplotype diversity of ND5 ranged between 0.818 (2010) and 0.861 (2011) while the nucleotide diversity (*π*) varied from 0.004 (2010) to 0.005 (2012) (Table 3). Pairwise *F*_ST_ comparisons did not reveal evidence of population structure based on mtDNA amongst cohorts, suggesting a stable pattern over the course of this study (highest *F*_ST 2010 vs. 2011_ = 0.0002, *P *=* *0.36). All sequences can be found in Appendix S1.

**Table 3:** Genetic diversity indices and condition index of each cohort

Cohort	*n*	Mitochondrial DNA				Microsatellites			
		*n*Hap	*S*	Hd	*π*	He	Ar	*F*_IS_	Kn
2010	155	31	30	0.818	0.004	0.734	14.52	0.159	0.944 (0.091)
2011	127	34	31	0.861	0.005	0.747	15.21	0.165	0.993 (0.114)
2012	121	34	35	0.851	0.005	0.748	15.40	0.166	1.061 (0.089)

*n *= number of individual analyzed. Mitochondrial data from a 355 base‐pair fragment of the ND5: *n*Hap = number of haplotypes, *S* = segregation sites, *π =* nucleotide diversity; Microsatellite data from 22 *loci*: He, heterozygosity; Ar, rarefied allelic richness; *F*_IS_, inbreeding coefficient; Phenotypic data Kn, condition index. Values in brackets represent standard deviation.

**Figure 2:**
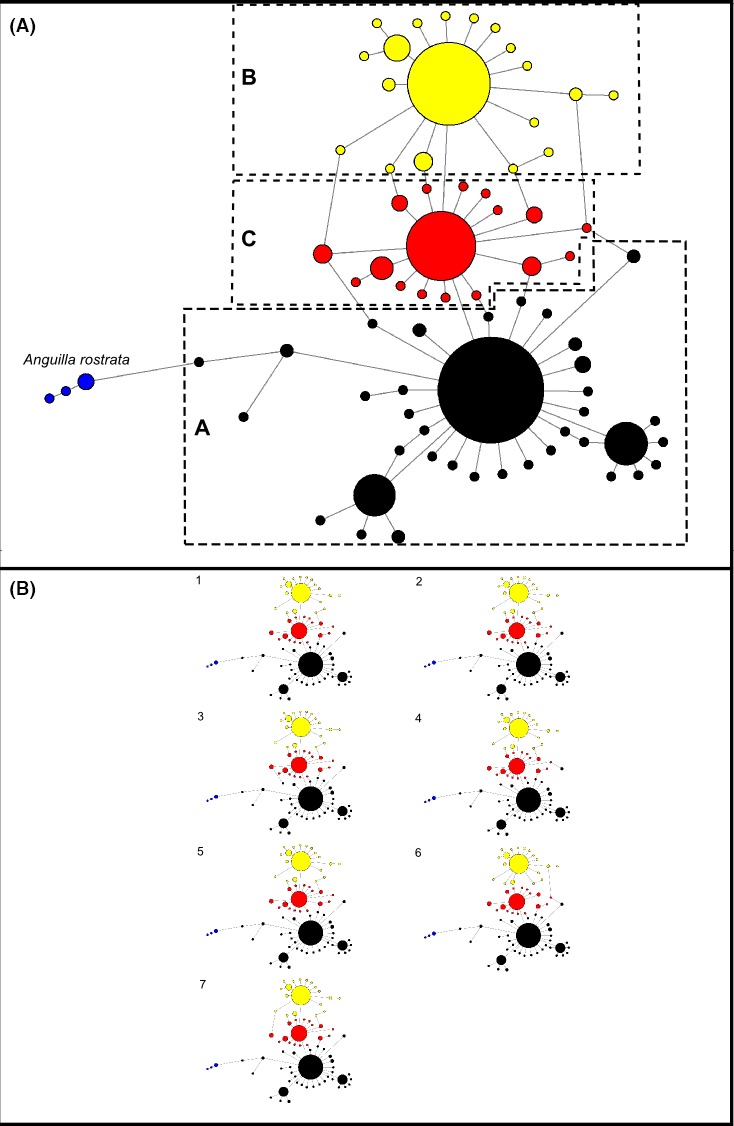
Main haplotype network and all shortest networks obtained through maximum parsimony. Panel (A) Network obtained with maximum parsimony approach already containing all possible trees. Letters correspond to matrilineages. The matrilines A, B and C are represented by colors black, yellow and red respectively. Blue represents *Anguilla rostrata* sequences added as an outgroup to provide a visual scale for differentiation. Mutation steps, median vectors and frequency of each haplotype can be consulted in Fig S1. Panel (B) All the seven possible shortest trees identified through maximum parsimony. The assignment of satellite haplotypes to respective matriline, that is A (black), B (yellow) and C (red), was performed through maximum parsimony analyses (Bandelt et al. 1999).

Reconstructing the *ND5* haplotype network revealed the existence of three major haplotypes and 68 satellite haplotypes, a structure similar to that of (Baltazar‐Soares et al. 2014; Fig. 2). Forty‐eight randomly picked singletons were verified by independent extraction, amplification and re‐sequencing to eliminate possible risks of sequencing errors. The three main haplotypes diverged from each other by one and two base pairs respectively. Noteworthy, this is to be compared with five *ND5* sequences of American eels which diverged from one European eel haplotype by as little as five base pairs (Fig. 2). The parsimonious resolution of connection ambiguities allowed us to delimitate three matrilines (see Fig. 2 and Fig. S1 for detailed information on the network). Each matriline consists of the main haplotype and respective satellites that directly connect to it. We named those matrilines “A”, “B” and “C” (Fig. 2). Matriline A was consistently observed as the most represented matriline accounting for 46–54% of the total number of individuals within cohorts (B ~ 30%, C ~ 20%).

#### Estimates of mutation rate, substitution model and demographic history of matrilines

Clock rate estimates based on the divergence time between *A. anguilla* and *A. rostrata* resulted in a mean mutation rate of 0.946 × 10^−5^ subs/site/million year (standard deviation = ±0.196 × 10^−5^). The substitution model HKY + I + G was selected amongst 24 others through Akaike information criteria (AIC) in jModeltest (AICc 1668.651, ΔAICc = 0) (Darriba et al. 2012). For the construction of the Bayesian skylines, we adopted the HKY + I + G substitution model. Also, the mean mutation rate was inserted as a uniform prior, with upper and lower boundaries representing mean ± standard deviation respectively.

**Figure 3:**
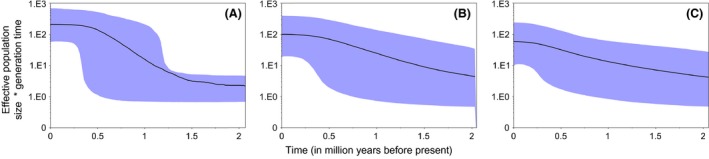
Matrilines demographic history. Bayesian skyline plots were constructed independently for each matriline. Here the *x*‐axis represents “time” and is defined in million years (my). *Y*‐axis represents the female *N*_e_ multiplied by generation time. The black line represents the median *N*_e_ and the shades the 95% high probability density interval.

Bayesian skyline plots revealed different patterns amongst matriline's demographic history: matriline A showed a steeper growth phase surrounded by two plateaus of constant sizes. The onset of the growth phase occurred ~1.5 million years ago (mya) and ended ~0.5 mya. This pattern contrasted with the single phase of constant growth that was observed in the demographic plots of matrilines B and C that extends throughout the historical timeline investigated (Fig. 3). Differences amongst demographic scenarios are also explicit in the distributions of the marginal probabilities of the run of each matriline, where each explored a different demographic space (Fig. S2). Noteworthy, all Markov chains reached convergence: the posterior's ESS was always above 200 (matriline A = 219; matriline B = 456; matriline C = 1249), with respective distributions of marginal likelihood probabilities proven to be unimodal (Fig. S2).

### Validation of microsatellite data: null alleles and selective neutrality

None of the 22 loci revealed a frequency of null alleles above the 20% threshold that is assumed to influence population differentiation estimates (Chapuis and Estoup 2007) (Table S1) – as estimated by Dempster's EM method (Dempster et al. 1977). Similarly, no alleles were fixed amongst loci. Selection tests also revealed that none of the 22 microsatellites loci deviated from a neutral mode of evolution (Fig. S3). This was true either when pairwise comparisons were performed between matrilines or amongst demes (Table S2). In addition, there was no correlation between mtDNA and microsatellite *F*_ST_ (Mantel test, *z* = 0.038, *P *=* *0.583). Consequently, all 22 microsatellite loci were kept for further analyses.

### Nuclear genetic diversities, contemporary demography and structure

When investigating cohort‐based indices of genetic diversity, that is allelic richness (Ar), Nei's unbiased heterozygosity (He) and *F*_IS_, we found that only the allelic richness Ar significantly differed amongst cohorts (ANOVA_Ar_: *F*_2_ = 7.01, *P *=* *0.03) with the 2012 cohort showing the highest level (mean Ar_2010_ = 9.780, SD = 0.130; mean Ar_2011_ = 10.073, SD = 0.161; mean Ar_2012_ = 10.117, SD = 0.005). Neither He nor *F*_IS_ significantly varied amongst cohorts (ANOVA_He_: *F*_2_ = 2.864, *P *=* *0.134; ANOVA_Fis_: *F*_2_ = 0.107, *P *=* *0.9). Focusing on matrilines, there were no differences observed for any of the tested indices (ANOVA_Ar_: *F*_2_ = 0.45, *P *=* *0.66; ANOVA_He_: *F*_2_ = 1.56, *P *=* *0.29; ${\text{ANOVA}}_{F_{\mathrm{IS}}}$: *F*_2_ = 0.40, *P *=* *0.68, Table 3 and Appendix S2). In relation to the individual‐based diversity indices (HL, IR), we found no differences amongst cohorts, amongst matrilines or amongst demes (ANOVA_HL_: cohorts *F*_2_ = 0.049, *P *=* *0.953; matrilines *F*_2_ = 0.315, *P *=* *0.730; demes *F*_8_ = 12.340, *P *=* *0.296; ANOVA_IR_: cohorts *F*_2_ = 0.001, *P *=* *0.996; matrilines *F*_2_ = 0.288, *P *=* *0.750; demes *F*_8_ = 10.219, *P *=* *0.396) (Table 4). BOTTLENECK tests revealed neither the occurrence of a mode shift in allele frequencies nor an excess of heterozygotes – the typical genetic signatures expected after a severe declines in population size (Table S3).

**Table 4:** Genetic diversity and condition index for each matriline within the cohorts

Matrilines	*n*	He	Ar	*F*_IS_	HL	IR	Kn
2010							
A	82	0.734	9.780	0.159	0.304 (0.101)	0.164 (0.123)	0.934 (0.086)
B	35	0.731	9.650	0.184	0.322 (0.091)	0.177 (0.117)	0.943 (0.100)
C	33	0.737	9.910	0.134	0.284 (0.097)	0.140 (0.119)	0.970 (0.089)
2011							
A	58	0.737	9.900	0.162	0.305 (0.108)	0.163 (0.132)	0.988 (0.108)
B	35	0.751	10.100	0.151	0.288 (0.104)	0.153 (0.121)	1.005 (0.122)
C	32	0.760	10.220	0.193	0.305 (0.089)	0.168 (0.118)	0.989 (0.119)
2012							
A	62	0.744	10.120	0.172	0.311 (0.097)	0.170 (0.110)	1.061 (0.090)
B	32	0.742	10.120	0.130	0.279 (0.124)	0.131 (0.150)	1.055 (0.090)
C	27	0.760	10.110	0.194	0.314 (0.094)	0.180 (0.117)	1.068 (0.090)

*n *= number of individuals analyzed, Microsatellite data from the 22 *loci*: He, heterozygosity; Ar, rarefied allelic richness; *F*_IS_, inbreeding coefficient; HL, homozygosity per loci; IR, internal relatedness; Phenotypic data from morphological measurements: Kn, condition index. Values in brackets represent standard deviation.

Pairwise *F*_ST_ comparisons showed low but statistically significant differentiation amongst cohorts after correction for false‐discovery rate (Narum 2006) (*F*_ST 2010/2011_ = 0.002, *P *=* *0.02). Similarly, when focusing on demes, four pairwise comparisons showed a *P*‐value lower than 0.05 and one remained statistically significant after correction for multiple testing (*F*_ST_ _2010A/2011B_ = 0.005, *P *=* *0.01, Table S4). The AMOVA considering “cohort” as the higher hierarchical group showed no statistical support (*F*_CT_ = 0.001, *P *=* *0.28).

Calculations of Evanno's Δ*K* (Evanno et al. 2005), and admixture plots in STRUCTURE revealed no clustering, as expected under the low *F*_ST_ observed (Table S5, Figs. S4 and S5).

### Comparing models: assessing the likelihood of structure and directionality of gene flow

To infer the likelihood of the matrilines to represent sub‐samples of a panmictic population, in contrast with the alternative hypothesis where each matriline would correspond to a reproductively isolated unit, we fitted our data into two population models and statistically tested which one would explain our data better.

**Figure 4:**
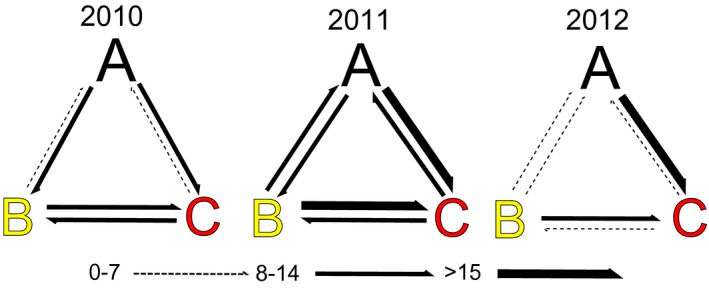
Effective number of migrants (*N*_em_) amongst demes in each cohort. Visual depiction of migration rates, or effective number of migrants (*N*_em_), estimated from the island model with bi‐directional gene flow simulated for each cohort. The arrows represent the flux, in terms of *N*_em_, and direction of migration. The categories of *N*_em_ are expressed in terms of thickness. *N*_em_ was calculated through *N*_em_ = (*Μ*_*j*_→_*i*_ *θ_I_)/4.

Average of the marginal log likelihoods of each model were calculated and compared to identify the most likely scenario (Beerli and Felsenstein 1999). Results showed that the model simulating a structured ground (model II) was favored over the model simulating a panmictic population (model I_MLog_ = −5400.113; model II_MLog_ = −3740.630, Bayes factor (model II_Mlog_ − model I_Mlog_) = 1659.483). Furthermore, results revealed the existence of asymmetric migration amongst matrilines. In particular, matriline A always acted as source for the others matrilines. This was clearly evident in the cohort of 2010, where emigration ranged between 10 and 12 *N*_em_, while immigration was reduced to <1 *N*_em_. In addition, for the 2012 cohort, emigration from deme A to deme C was the highest observed in this study, 21 *N*_em_, and contrasted with the immigration of <1 *N*_em_ (Table 5 and Fig. 4). Modes and confidence intervals of *θ* and *N*_em_ within each cohort can be found in Tables S6–S8. Posterior distributions of migration parameters are available in Figure S6.

**Table 5:** Mutation scaled effective population sizes (*θ*) and effective number of migrants (*N*_em_) of model II

	2010	2011	2012
*θ*A	0.067	2.600	0.067
*θ*B	2.067	2.067	0.067
*θ*C	2.467	4.600	4.200
*N*_em_ B→A	0.325	14.084	0.295
*N*_em_ C→A	0.318	12.350	0.328
*N*_em_ A→B	10.161	11.195	0.295
*N*_em_ C→B	8.095	8.738	0.195
*N*_em_ A→C	11.716	21.850	20.650
*N*_em_ B→C	9.661	19.550	12.250

*N*_em_ was calculated through *N*_em_ = (*Μ*_*j*_→_*i*_ *θ_I_)/4 for each deme.

### Impacts of gene flow on the genetic diversity of matrilines

Differences between correlation coefficients *R*_IR_ (correlation coefficient between mean IR and *F*_IS_) and *R*_HL_ (correlation coefficient between mean HL and *F*_IS_) are particularly informative in the presence of migration: under asymmetric gene flow amongst demes, one would expect *R*_HL_ **> *R*_IR_ if immigration would bring new or low frequency alleles to the receiving demes. Here, we detected a coefficient *R*_IR_ of 0.93 (*P *<* *0.001) and a coefficient *R*_HL_ of 0.88 (*P *=* *0.002). The incorporation of HL and IR as independent explanatory variables in a linear model confirmed that IR explains a higher proportion of *F*_IS_ than HL (*t*_IR_ **= 2.380, *P *=* *0.06; *t*_HL_ **= −0.543, *P *=* *0.61).

### Heterozygosity‐fitness correlations

To explore potential drivers of the variations in condition index (Kn) observed amongst cohorts or amongst demes we fitted a linear model where we included potential effects of mitochondrial lineage (mtDNA) and individual diversity indices such as HL and IR. We found that Kn varied only amongst cohorts (*F*_2_ = 48,654, *P *<* *0.001, Table 6).

**Table 6:** Effects of possible explanatory variables to condition index variation

Kn*^˜^*	df	*F*	*P*
Rds	1	3.714	0.055
IR	1	0.235	0.628
Cohort	2	48.654	<0.001*
Rds:IR	1	0.048	0.828
Cohort:matriline	6	0.653	0.688
Rds:cohort	2	1.104	0.333
IR:cohort	2	1.237	0.292
Rds:cohort:matriline	6	1.891	0.082
IR:cohort:matriline	6	1.114	0.354
Rds:IR:cohort	2	0.585	0.558
Rds:IR:cohort:matriline	6	0.990	0.432

Rds, residuals of the correlation of HL and IR; mtDNA, matriline; *represents a significant effect.

## Discussion

Due the many challenges associated with research expeditions, understanding the biology and evolution of the European eel still strongly relies on indirect genetic evidence. In this study, we undertook the task of comparing the genetic signature of a homogenous spawning ground, where reproduction would be panmictic, with the signature of a hypothetical matriline‐driven structured spawning ground.

To do so, we screened for the occurrence of the three major haplotypes groups investigated in a previous study across three consecutive cohorts of glass eels (Baltazar‐Soares et al. 2014). The underlying goal was to provide theoretical explanation to the previously observed structure on the European coasts. To this end, we grouped individuals according to their matrilines and then used nuclear markers to evaluate possible gene flow. The comparison of the models representing panmixia and a structured population linked to matrilines revealed better support for the data to be explained by female segregated spawning units. Surprisingly, inferences of gene flow amongst matrilines revealed that migration is asymmetric, inconsistent with a panmictic mode of reproduction. Our study thus points towards the existence of female‐mediated reproductive units, which we speculate its detection to be a consequence of a fragmented spawning ground due to the lasting low recruitment that this species is experiencing.

### Definition, historical demography and contemporary structure of matrilines

By constructing a haplotype network based on the ND5 mitochondrial gene, we confirmed the existence of a stable haplotype network structure, dominated by three major haplotypes separated by 1–2 base pairs. As these differences are in line with site‐specific haplotype differences in philopatric species (Keeney et al. 2005; Levin and Parker 2012), we grouped our samples accordingly and assumed it to represent female‐mediated reproductive units. Upon their definition, we investigated the demographic history of each matriline. This scenario is conceptually plausible as the recruitment collapse that the European eel has undergone may have fragmented its spawning ground isolating reproductive demes, as is often the case with declining population size (Yamamoto et al. 2004; Hastings and Botsford 2006).

Skyline plots suggest that all matrilineages are likely experiencing an historical growth phase yet with contrasting shapes: the most common matriline A displays a steeper and more pronounced expansion compared to the two others. This expansion has started ~1.25 mya and lasted for ~1 my, and can be justified by several hypotheses. First, it may relate to a major geological event matching a high velocity phase of the Gulf Stream's western boundary current (Kaneps 1979). This is a possible explanation given the key role of this major current in the contemporary dynamics of the European eel population (Bonhommeau et al. 2009; Blanke et al. 2012). Second, it may be a gene‐specific signature. Screening the full mitogenome (Jacobsen et al. 2014a) found a past decline and recovery of the eel population. Mitogenome analyses provide robust inference of the historical demography with higher resolution than a single gene. Furthermore, simulations performed by (Grant et al. 2014) suggested that Bayesian skylines applied to a single mitochondrial gene may only capture the most recent event of a population expansion. Thus, our ND5‐based skyline plots might only be capturing the recent expansion detected by (Jacobsen et al. 2014a). However, like previous studies (Pujolar et al. 2011; Jacobsen et al. 2014a), we did not capture the signature of a recent decline.

### Measures of gene flow: insights into the structure and dynamics of the European eel population

The definition of matrilines allowed us to test for the statistical robustness of a panmictic mode of reproduction versus segregated units of matrilines based on gene flow estimated with 22 microsatellites. We used Bayesian statistics coupled with coalescent theory, a framework that is becoming increasingly acknowledged as an ideal inferential approach to test connectivity amongst putative populations (Beerli and Palczewski 2010). Quite surprisingly, results support the hypothesis that genetic diversity is best explained by a structured spawning ground rather than a panmictic mode of reproduction (Bayes factor: model II_Mlog_ − model I_Mlog_ = 1659.483). We are fully aware that this methodology does not provide final proof regarding the true structure of a population (Beerli and Palczewski 2010). The high acceptance rate of the harmonic mean estimator in identifying panmixia (70%) in a true panmictic population (Beerli and Palczewski 2010) suggests, however, that panmixia is less likely to be the underlying structure observed in our samples.

Interestingly, in addition to the support for the model assuming a segregated spawning ground, we detected asymmetry of the migration rates amongst the matrilines. Particularly, over the three sampled cohorts, the most common matriline always acted as a source of migrants for the other matrilines. While consistent asymmetries in migration rates are not predicted under panmixia (Waples and Gaggiotti 2006), they are commonly reported amongst philopatric demes in highly migratory species with sex‐biased dispersal such as sperm whales (Lyrholm et al. 1999), salmonids (Fraser et al. 2004), or turtles (Stiebens et al. 2013). It is thought to reflect the contrast between opportunistic mating of one of the sexes and faithfulness to specific spawning conditions of the other. Given that we grouped samples by matriline, the asymmetries reported here should reflect male‐mediated gene flow.

One critical insight is gained from these observations: asymmetries in gene flow can generate low but significant *F*_ST_ amongst the putative demes (0.001 < *F*_ST_ < 0.006). While differentiation estimates of this order of magnitude may escape the scrutiny of Bayesian clustering methods (Pearse and Crandall 2004; Anseeuw et al. 2008), it is interesting to note that those values and significance are extremely similar to those reported by (Dannewitz et al. 2005) or (Pujolar et al. 2009a) but differ in their interpretations in the light of a female‐mediated structure (Baltazar‐Soares et al. 2014). To exist, gene flow among female‐driven reproductive units should be male‐mediated. This assumption is coherent with the speculative life‐history of male European eels, who are believed to mature before the females, consequently returning to the spawning grounds faster and thus mixing cohorts (Tesch 2003).

We uphold the suggestion that the results can be interpreted in light of the recruitment collapse that occurred in the early 1980s. The lack of spawners associated with the collapse (Dekker 2003; Aalto et al. 2016) may have led to a fragmentation of the spawning area in the Sargasso Sea creating semi‐isolated reproductive demes. The detection of genetic structure due to habitat fragmentation is a well described phenomenon (Monaghan et al. 2002; Yamamoto et al. 2004; Hastings and Botsford 2006). Our data suggest that under high abundance of spawners, female‐mediated patterns may not be detected because of leakage at the margins of the units. However, under reduced numbers of mature fish, this structure is observed. If this structure was to be verified, it would explain why despite a drastic recruitment collapse down to <10% of historical record, no genetic signature of bottleneck was observed (Pujolar et al. 2011 and this study). Indeed, male‐mediated gene flow would allow the transport of alleles among demes at each reproductive event maintaining the genetic variability, as well as the adaptive potential currently observed in the population.

Lastly, there is also the possibility for *F*_ST_ and gene flow asymmetries here reported using microsatellites to be associated with introgressive hybridization between *A. anguilla* and *A. rostrata* (Pujolar et al. 2014a; Wielgoss et al. 2014). It has been shown through simulations that variable admixture rates, in the Sargasso Sea, can produce a latitudinal cline on *F*_ST_ differentiation similar to a pattern of isolation by distance, across the European coast (Wielgoss et al. 2014). We cannot discard a possible role for hybridization, despite such patterns have not been reported to low latitudes as was our sampling site.

### Heterozygosity‐fitness correlations: linking demography and evolution?

To infer whether different matrilines are associated with variable fitness traits, we performed heterozygosity‐fitness correlations using body condition post‐transatlantic migration as fitness trait of interest. Assuming that female philopatry evolved to maximize successful transport of offspring under given oceanic conditions (Baltazar‐Soares et al. 2014), lineage‐specific fitness would be a reasonable expectation. Variable recruitment could be related to variation in condition index amongst the different matrilines, linking recruitment and lineage‐specific fitness. However, we observed that “cohort” is the major effect determining condition index variation. This means that either the condition index upon arrival is not directly related to matriline fitness, or, more likely that variation in the condition index is linked to ocean‐mediated recruitment success (Friedland et al. 2007; Bonhommeau et al. 2008; Kettle et al. 2008; Melià et al. 2013; Baltazar‐Soares et al. 2014). For example, if ocean currents promote a faster transatlantic migration of the 2012 cohort (in comparison with 2010) those individuals would have consumed less internal nutritional reserves and therefore achieved higher condition index at arrival.

## Conclusions

Because direct sampling of adult spawners in the Sargasso Sea is a challenge yet to be overcome, to understand the evolution of the European eel, new theories have to be proposed and tested. Here, we built on previous modelling work which suggested that a female structured spawning ground could explain the punctual reports of genetic structure in a population otherwise considered panmictic. Artificially creating those female philopatric groups and testing for the population signature against panmixia suggested that multiple (in time or space) spawning grounds may exist.

Rather than bringing conclusive evidence for the existence of a structured European eel spawning ground, the work we here present aimed at opening avenues of research on the much challenging theme of eel population genetics. We are greatly aware of the limits of the study, which primarily relies on strong assumptions associated with the definition of matrilines. Further studies challenging the understanding of the mode of evolution of this enigmatic species could, for instance, expand the scope using complete mitogenome or genome‐wide markers (Jacobsen et al. 2014b; Pujolar et al. 2014b) to hypothesize variable scenarios and test for their possible existence.

## Conflict of Interest

None declared.

## Supporting information


**Figure S1.** Haplotype network, with all shortest trees considered, with explicit mutation steps and frequencies of each haplotype >2. The coor code for each matriline is the following: A = Black, B = Yellow and C = Red.

**Figure S2**. Posterior's marginal likelihood probability distributions of the BEAST runs for each matrilineage. The *x*‐axis represents the posterir while the *y*‐axis represents the density, or the explored parameter space. The effective sample sizes (ESS) of the posterior parameter of each run were as following: A = 219, B = 456, C = 1249. These plots were produced in Tracer (Rambaut 2014).

**Figure S3**. Graphical display of the simulated confidence areas for each of the respective modes of evolution. Blue dots and respective labels correspond to the markers used in this study. This pattern is common to the infinite allele and stepwise mutation modes of evolution and shows all loci behaving as candidate neutral. The *x*‐axis depicts the expected heterozigosity (He) while the *y*‐axis the *F*_ST_.

**Figure S4**. Evanno's *ΔK* calculated has (*ΔK* = mean(|L''(*K*)|)/sd(L(*K*))(Evanno et al. 2005). The *y*‐axis represents *ΔK* from *K* = 2 to *K* = 9 (*x*‐axis). The modal value of the distribution is the most likely number of clusters. Although peaks were observed in *K* = 2 and *K* = 4 , it is worth mentioning that the Evanno's method cannot detect *K* = 1 (Evanno et al. 2005).

**Figure S5**. STRUCTURE admixture plots for the modal distributions of *K* = 2 and *K* = 4 identified as possible *K*'s after (Evanno et al. 2005). Symmetry across both plots suggests that *K* = 1 is the most likely number of *K*.

**Figure S6**. Posterior distributions of migrations rates summed over all the loci for each cohort. The direction of migration is shown with the symbol ‐> , while the numbers “1”, “2” and “3” correspond to the matrilineages “A”, “B” and “C” respectively.

**Table S1.** Estimates of null allele frequencies for each locus obtained with Dempster's EM method [1]. Confidence intervals are given.
**Table S2.** Neutrality test on microsatellite data.
**Table S3.** Results of demographic analyses on microsatellites
**Table S4.** *F*_ST_ values for mtDNA (below diagonal) and microsatellites (above diagonal) considering the 9 demes.
**Table S5.** Outputs of the four‐step process following Evanno et al. to calculate ΔK [2].
**Table S6.** Estimates for the modes and respective 95% confidence interval for mutation‐scaled effective population size (*θ*) and *N*_em_ in model II, where 4 *N*_em_ = *Mj*→_i_*θ for 2010.
**Table S7.** Estimates for the modes and respective 95% confidence interval for mutation‐scaled effective population size (*θ*) and *N*_em_ in model II, where 4 *N*_em_ = *M*_*j*_→i*θ for 2011.
**Table S8.** Estimates for the modes and respective 95% confidence interval for mutation‐scaled effective population size (*θ*) and *N*_em_ in model II, where 4 *N*_em_ = *M*_*j*_→i*θ for 2012.

**Appendix S1.** Mitochondrial DNA sequences (ND5).

**Appendix S2.** Microsatellite allelic frequencies.
