# Supplementary figures and images for: Does asymmetric gene flow among matrilines maintain the evolutionary potential of the European eel?
Source: Ecol Evol. 2016 Jun 30;6(15):5305–20. doi: 10.1002/ece3.2098 (PMC4984505; doi:10.1002/ece3.2098)

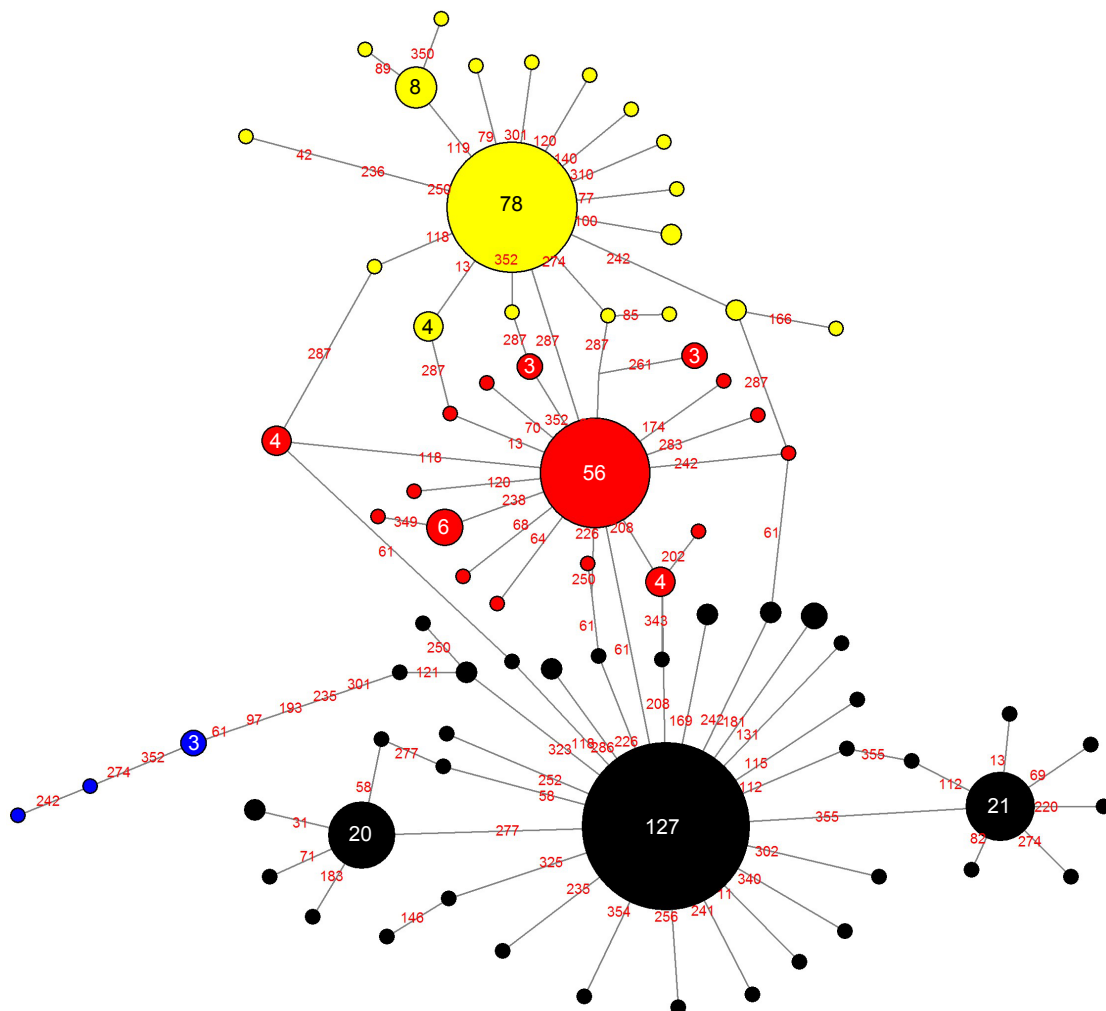

Supplement: Supplementary file 1 — Figure S1. Haplotype network, with all shortest trees considered, with explicit mutation steps and frequencies of each haplotype >2. The coor code for each matriline is the following: A = Black, B = Yellow and C = Red. [file ECE3-6-5305-s001.pdf]

Posterior's marginal probability distribuiton

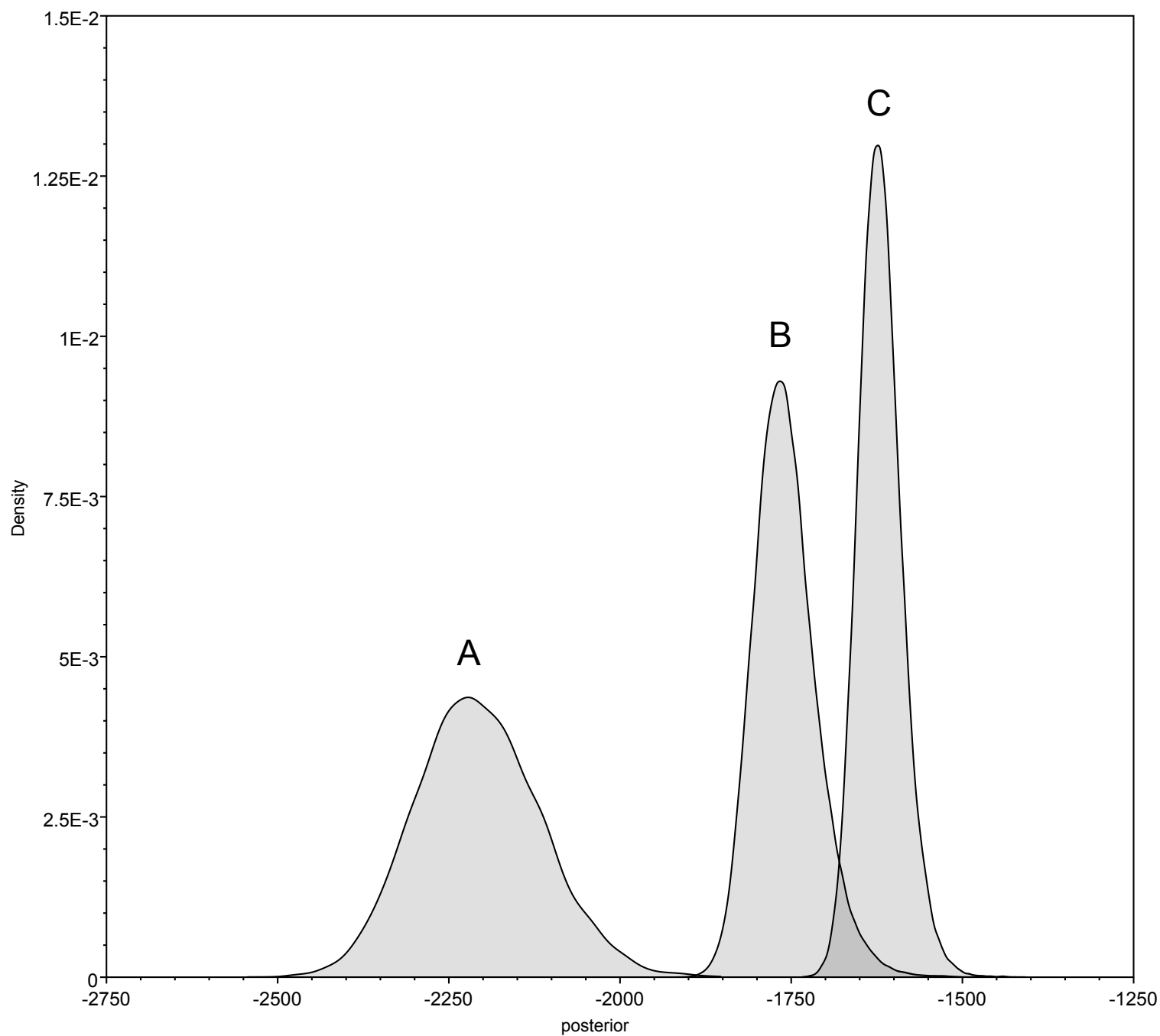

Supplement: Supplementary file 2 — Figure S2. Posterior's marginal likelihood probability distributions of the BEAST runs for each matrilineage. The x‐axis represents the posterir while the y‐axis represents the density, or the explored parameter space. The effective sample sizes (ESS) of the posterior parameter of each run were as following: A = 219, B = 456, C = 1249. These plots were produced in Tracer (Rambaut 2014). [file ECE3-6-5305-s002.pdf]

# Fst/He

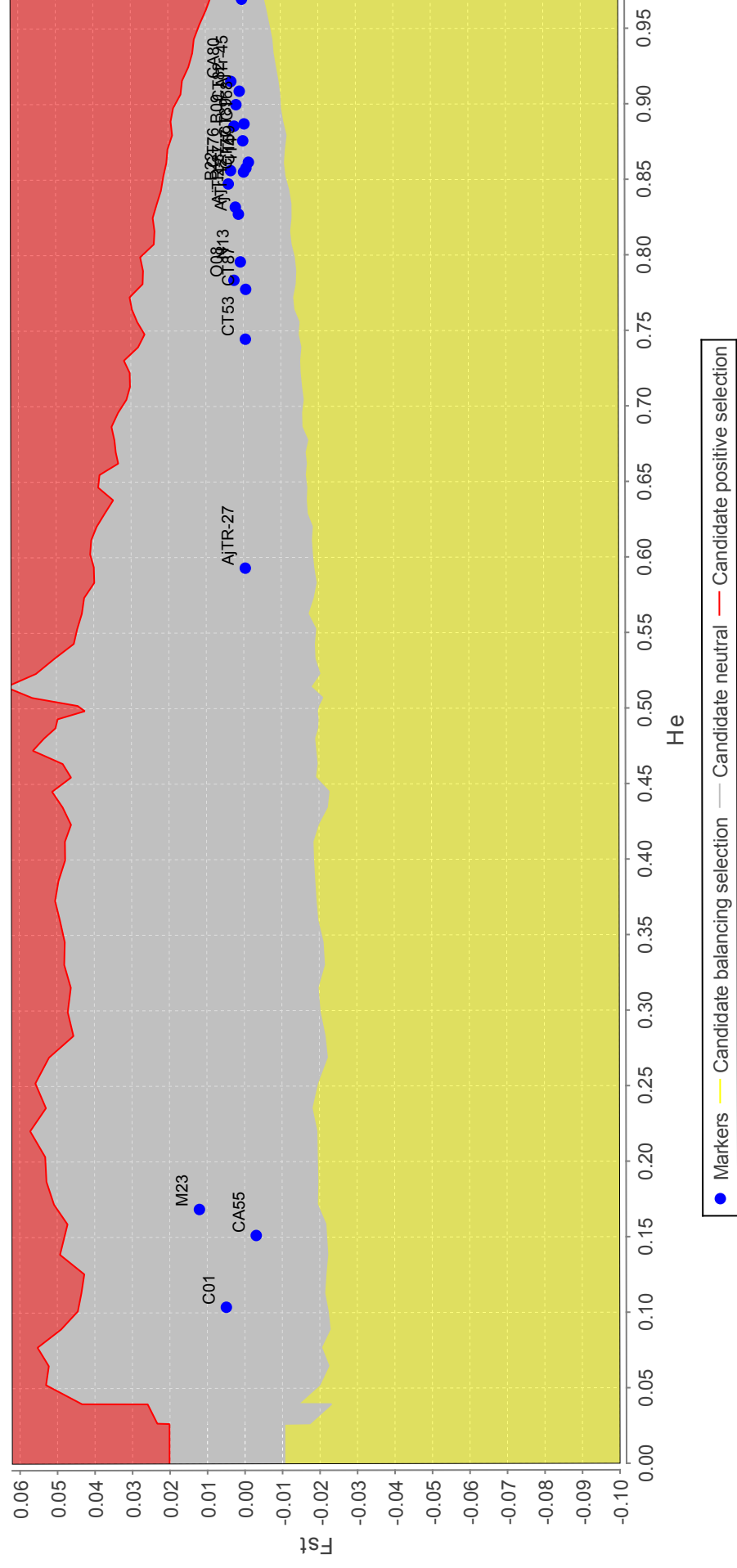

Supplement: Supplementary file 3 — Figure S3. Graphical display of the simulated confidence areas for each of the respective modes of evolution. Blue dots and respective labels correspond to the markers used in this study. This pattern is common to the infinite allele and stepwise mutation modes of evolution and shows all loci behaving as candidate neutral. The x‐axis depicts the expected heterozigosity (He) while the y‐axis the F ST. [file ECE3-6-5305-s003.pdf]

$$\text{DeltaK} = \text{mean}(|L''(K)|) / \text{sd}(L(K))$$

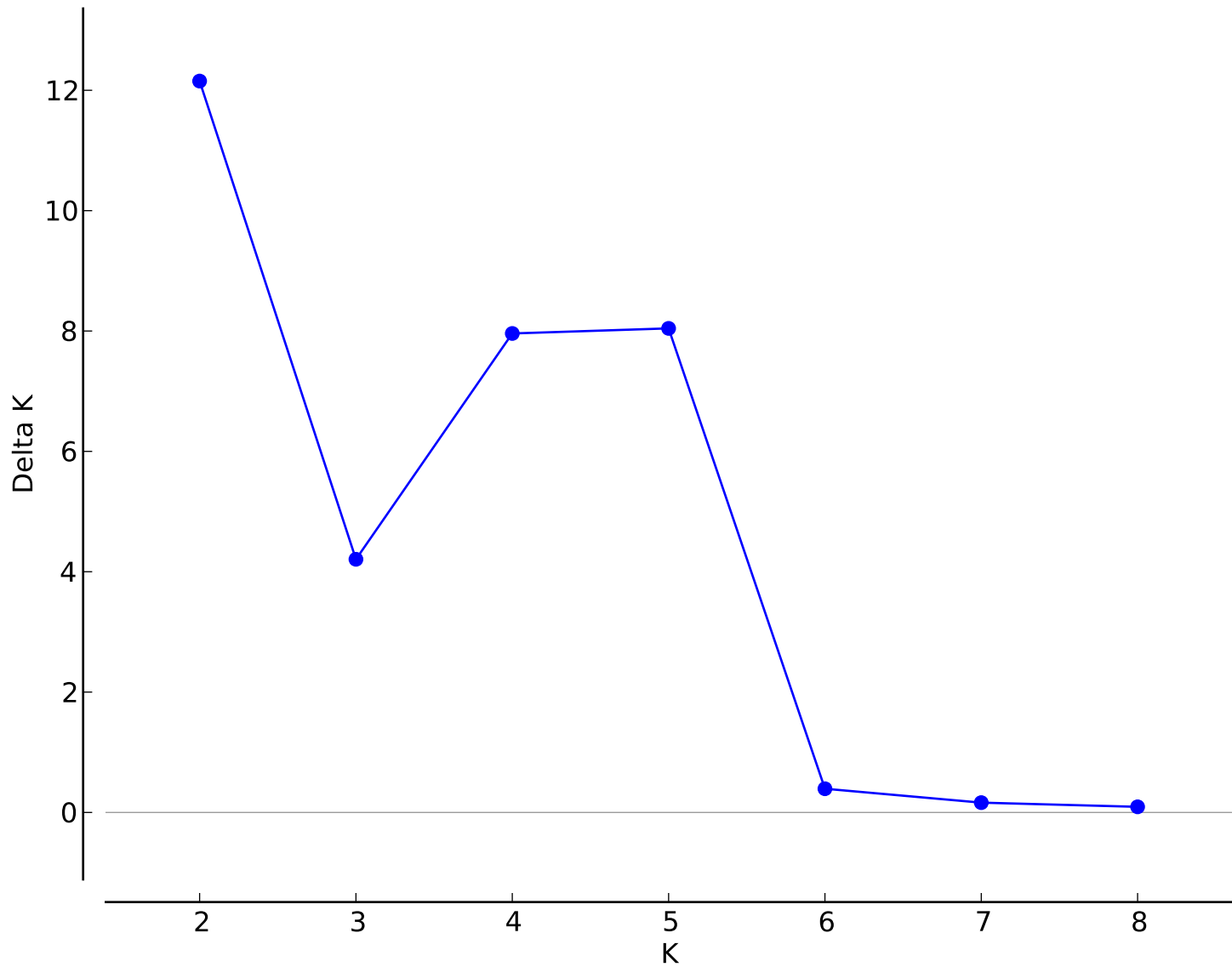

Supplement: Supplementary file 4 — Figure S4. Evanno's ΔK calculated has (ΔK = mean(|L''(K)|)/sd(L(K))(Evanno et al. 2005). The y‐axis represents ΔK from K = 2 to K = 9 (x‐axis). The modal value of the distribution is the most likely number of clusters. Although peaks were observed in K = 2 and K = 4 , it is worth mentioning that the Evanno's method cannot detect K = 1 (Evanno et al. 2005). [file ECE3-6-5305-s004.pdf]

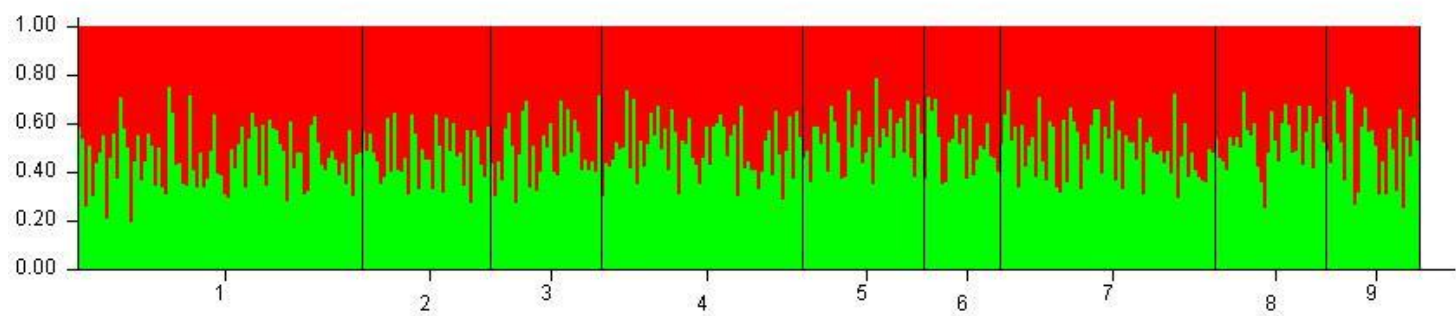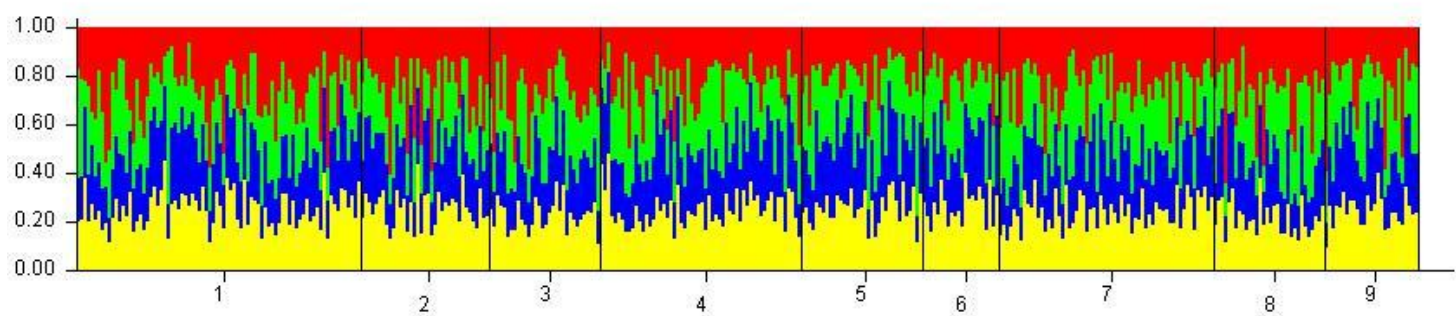

Supplement: Supplementary file 5 — Figure S5. STRUCTURE admixture plots for the modal distributions of K = 2 and K = 4 identified as possible K's after (Evanno et al. 2005). Symmetry across both plots suggests that K = 1 is the most likely number of K. [file ECE3-6-5305-s005.pdf]

# 2010

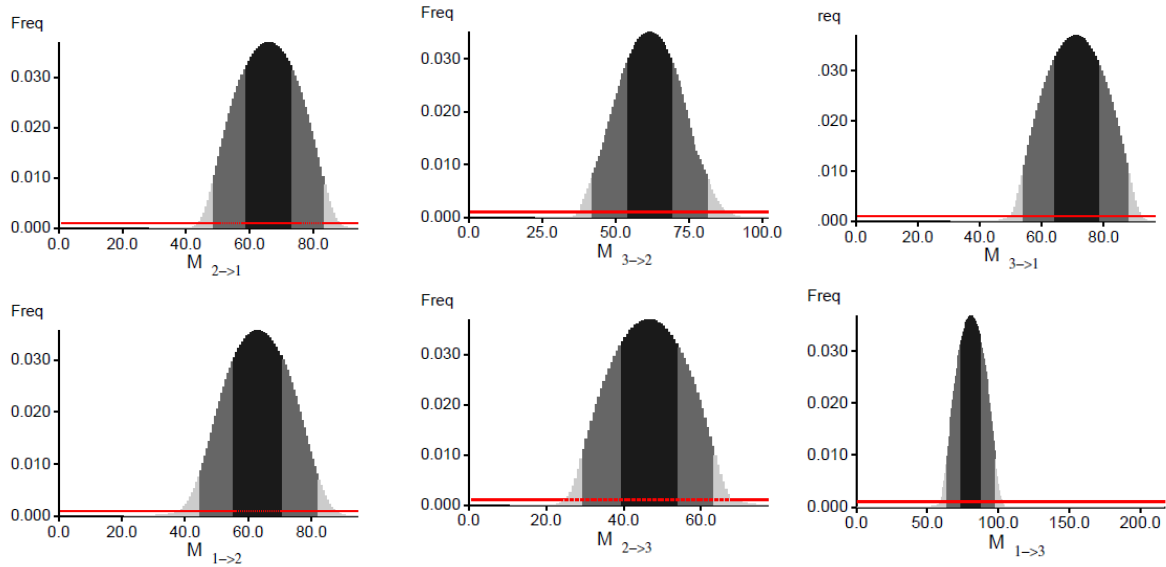

# 2011

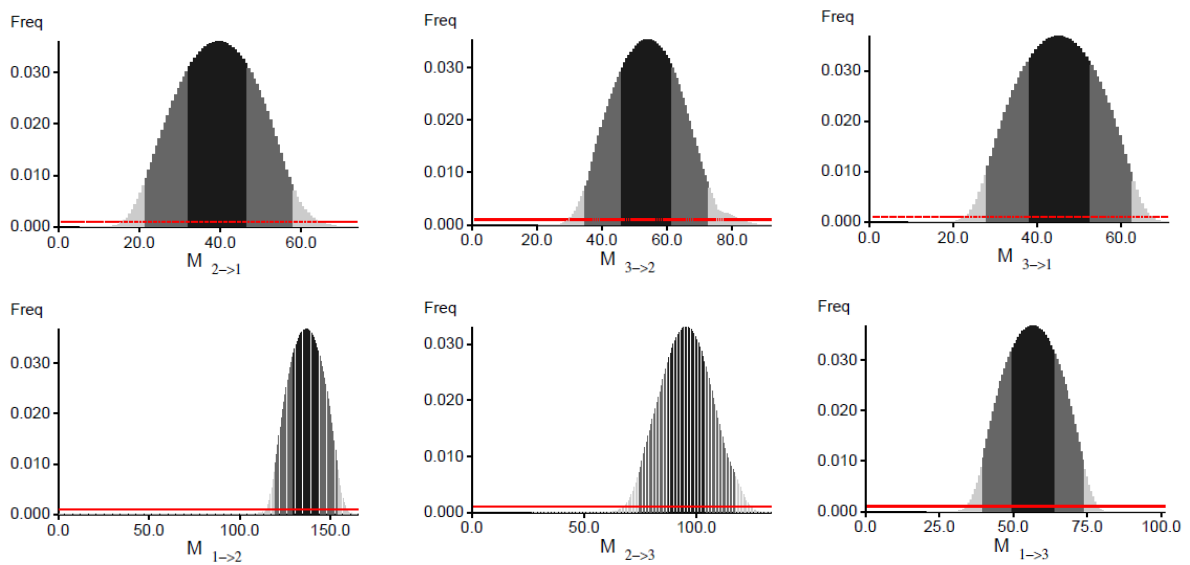

# 2012

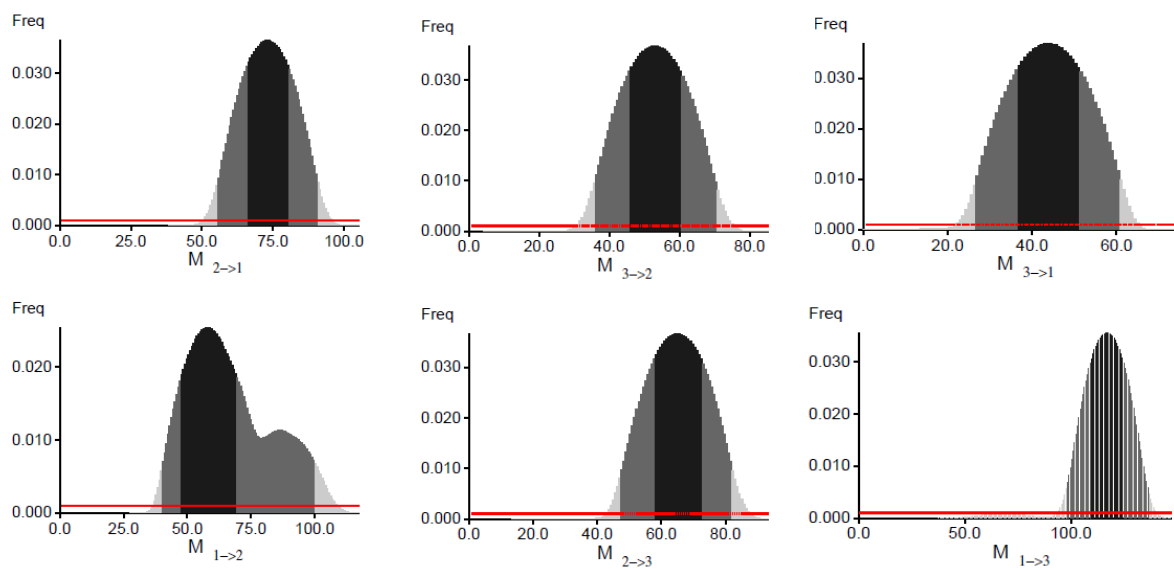

Supplement: Supplementary file 6 — Figure S6. Posterior distributions of migrations rates summed over all the loci for each cohort. The direction of migration is shown with the symbol ‐> , while the numbers “1”, “2” and “3” correspond to the matrilineages “A”, “B” and “C” respectively. [file ECE3-6-5305-s006.pdf]
