# Supplementary material for: Does asymmetric gene flow among matrilines maintain the evolutionary potential of the European eel?
Source: Ecol Evol. 2016 Jun 30;6(15):5305–20. doi: 10.1002/ece3.2098 (PMC4984505; doi:10.1002/ece3.2098)
Supplement: Supplementary file 7 — Table S1. Estimates of null allele frequencies for each locus obtained with Dempster's EM method [1]. Confidence intervals are given. Table S2. Neutrality test on microsatellite data. Table S3. Results of demographic analyses on microsatellites Table S4. F ST values for mtDNA (below diagonal) and microsatellites (above diagonal) considering the 9 demes. Table S5. Outputs of the four‐step process following Evanno et al. to calculate ΔK [2]. Table S6. Estimates for the modes and respective 95% confidence interval for mutation‐scaled effective population size (θ) and N em in model II, where 4 N em = Mj→i*θ for 2010. Table S7. Estimates for the modes and respective 95% confidence interval for mutation‐scaled effective population size (θ) and N em in model II, where 4 N em = M j→i*θ for 2011. Table S8. Estimates for the modes and respective 95% confidence interval for mutation‐scaled effective population size (θ) and N em in model II, where 4 N em = M j→i*θ for 2012. [file ECE3-6-5305-s007.docx]

**Supplementary Tables and text**

**Supplementary tables**

Table S1 – Estimates of null allele frequencies for each locus obtained with Dempster’s EM method [1]. Confidence intervals are given.

| Locus | Frequency Estimate | 2.5% | 97.5% |
| --- | --- | --- | --- |
| CT77 | 0.0931 | 0.0698 | 0.1192 |
| CT68 | 0.0247 | 0.0102 | 0.0448 |
| CA58 | 0.0720 | 0.0546 | 0.0931 |
| CT87 | 0.0712 | 0.0487 | 0.0977 |
| AjTR-37 | 0.0364 | 0.0189 | 0.0590 |
| CA55 | 0.0722 | 0.0380 | 0.1179 |
| CT59 | 0.0875 | 0.0652 | 0.1131 |
| CT76 | 0.0521 | 0.0320 | 0.0763 |
| CT53 | 0.0100 | 0.0000 | 0.0326 |
| CT89 | 0.0413 | 0.0226 | 0.0641 |
| CA80 | 0.1283 | 0.1050 | 0.1543 |
| CT82 | 0.0292 | 0.0135 | 0.0496 |
| I14 | 0.0359 | 0.0178 | 0.0587 |
| AjTR-27 | 0.1708 | 0.1394 | 0.2039 |
| O08 | 0.0255 | 0.0080 | 0.0487 |
| M23 | 0.0629 | 0.0319 | 0.1003 |
| AjTr-45 | 0.1629 | 0.1356 | 0.1935 |
| C01 | 0.0567 | 0.0247 | 0.0948 |
| N13 | 0.1016 | 0.0774 | 0.1287 |
| B22 | 0.1601 | 0.1322 | 0.1909 |
| AjTr-42 | 0.1654 | 0.1370 | 0.1965 |
| B09 | 0.1167 | 0.0930 | 0.1441 |

Table S2 - Neutrality test on microsatellite data

| **Locus** | **He** | **F_ST_** | **p-value** |
| --- | --- | --- | --- |
| CT77 | 0.855186 | -0.000144 | 0.521398 |
| CT68 | 0.887046 | -0.000276 | 0.506718 |
| CA58 | 0.969604 | 0.00033 | 0.476317 |
| CT87 | 0.777514 | -0.000657 | 0.50849 |
| AjTR-37 | 0.831934 | 0.002066 | 0.621195 |
| CA55 | 0.15116 | -0.003071 | 0.482494 |
| CT59 | 0.857765 | -0.000776 | 0.494237 |
| CT76 | 0.856145 | 0.003326 | 0.665974 |
| CT53 | 0.7445 | -0.000557 | 0.536052 |
| CT89 | 0.875864 | 6.2e-05 | 0.515203 |
| CA80 | 0.915193 | 0.003215 | 0.668654 |
| CT82 | 0.899762 | 0.001866 | 0.594884 |
| I14 | 0.861668 | -0.001434 | 0.440095 |
| AjTR-27 | 0.592935 | -0.000439 | 0.569804 |
| O08 | 0.783527 | 0.002463 | 0.605455 |
| M23 | 0.168427 | 0.012069 | 0.759177 |
| AjTr-45 | 0.908689 | 0.000975 | 0.561676 |
| C01 | 0.103681 | 0.00493 | 0.684484 |
| N13 | 0.795682 | 0.000754 | 0.569096 |
| B22 | 0.847296 | 0.003938 | 0.68385 |
| AjTr-42 | 0.827263 | 0.001252 | 0.583101 |
| B09 | 0.885558 | 0.002383 | 0.634833 |

F_ST_ / expected heterozygosity (He) relationship as well as p-value assuming neutral evolution in an island model with migration and considering 9 populations. ***** denotes significant p-values (0.05) suggesting positive or balancing selection.

Table S3 – Results of demographic analyses on microsatellites

|  | ***2010*** | | | ***2011*** | | | ***2012*** | | |
| --- | --- | --- | --- | --- | --- | --- | --- | --- | --- |
| *Matriline* | *A* | *B* | *C* | *A* | *B* | *C* | *A* | *B* | *C* |
| *Allele frequency class* | *Frequencies* | | | | | | | | |
| 0.1 | 0.789 | 0.738 | 0.742 | 0.797 | 0.736 | 0.711 | 0.780 | 0.738 | 0.734 |
| 0.2 | 0.137 | 0.169 | 0.164 | 0.129 | 0.155 | 0.180 | 0.143 | 0.167 | 0.154 |
| 0.3 | 0.033 | 0.048 | 0.049 | 0.031 | 0.081 | 0.066 | 0.040 | 0.048 | 0.066 |
| 0.4 | 0.020 | 0.020 | 0.020 | 0.017 | 0.000 | 0.013 | 0.017 | 0.024 | 0.025 |
| 0.5 | 0.003 | 0.008 | 0.008 | 0.010 | 0.016 | 0.009 | 0.007 | 0.004 | 0.004 |
| 0.6 | 0.007 | 0.000 | 0.000 | 0.003 | 0.000 | 0.009 | 0.000 | 0.008 | 0.004 |
| 0.7 | 0.000 | 0.004 | 0.004 | 0.000 | 0.000 | 0.000 | 0.003 | 0.000 | 0.000 |
| 0.8 | 0.000 | 0.000 | 0.000 | 0.000 | 0.000 | 0.000 | 0.000 | 0.000 | 0.000 |
| 0.9 | 0.000 | 0.000 | 0.000 | 0.000 | 0.000 | 0.013 | 0.007 | 0.000 | 0.008 |
| 1 | 0.010 | 0.012 | 0.012 | 0.010 | 0.012 | 0.000 | 0.003 | 0.012 | 0.004 |
| *Probability*  *H deficiency* | 0.50000 | 0.61274 | 0.67218 | 0.30508 | 0.84735 | 0.78776 | 0.25142 | 0.71697 | 0.38726 |
| *Probability*  *H excess* | 0.51269 | 0.39952 | 0.33943 | 0.70604 | 0.16044 | 0.22170 | 0.75870 | 0.29396 | 0.62488 |
| *Probability H excess or deficiency* | 1.00000 | 0.79903 | 0.67886 | 0.61015 | 0.32088 | 0.44341 | 0.50284 | 0.58793 | 0.77453 |
| *Frequency distribution* | Normal L-shaped | Normal L-shaped | Normal L-shaped | Normal L-shaped | Normal L-shaped | Normal L-shaped | Normal L-shaped | Normal L-shaped | Normal L-shaped |

Inferences on allele frequency shifts and heterozygote excess/deficiency of each deme within cohorts Wilcoxon probability tests were made to test for heterozygote deficiency, excess (one tail test) and for excess or deficiency (two tail tests). Significant comparisons are marked with *. BOTTELNECK runs were performed (Cornuet & Luikart 1996) performed under a two-phase model of evolution (TPM), considering a 10% stepwise mutation and a 10% variance for the TPM.

Table S4 – F_ST_ values for mtDNA (below diagonal) and microsatellites (above diagonal) considering the 9 demes.

|  | A2010 | B2010 | C2010 | A2011 | B2011 | C2011 | A2012 | B2012 | C2012 |
| --- | --- | --- | --- | --- | --- | --- | --- | --- | --- |
| A2010 | 0 | 0.001 | 0.003 | 0.003 | 0.005** | 0,003 | 0,002 | 0,004 | 0,002 |
| B2010 | 0,704** | 0 | 0,001 | 0,002 | 0,004 | 0,001 | 0,001 | 0,000 | 0.003 |
| C2010 | 0.577** | 0.542** | 0 | 0.004 | 0.005 | 0.001 | 0.001 | 0.000 | 0.003 |
| A2011 | -0.003 | 0.638** | 0.501** | 0 | 0.004 | 0.003 | 0.004* | -0.002 | 0.003 |
| B2011 | 0.714** | -0.012 | 0.563** | 0.648** | 0 | 0.000 | 0.005* | 0.004 | 0.007* |
| C2011 | 0.574** | 0.533** | 0.012 | 0.492** | 0.555** | 0 | 0.001 | 0.000 | -0.002 |
| A2012 | -0.005 | 0.682** | 0.550** | -0.001 | 0.691** | 0.543** | 0 | 0.002 | 0.000 |
| B2012 | 0.727** | -0.004 | 0.582** | 0.660** | -0.003 | 0.573** | 0.704** | 0 | 0.006 |
| C2012 | 0.554** | 0.500** | -0.011 | 0.475** | 0.520** | 0.007 | 0.521** | 0.534** | 0 |

***** and ****** denotes p-values significant for 0.05 and 0.01 (after correction for false discovery rate (Narum 2006). Noteworthy, the high values of mtDNA are a by-product of the separation of the samples based on mtDNA haplotypes, and were only calculated to investigate the relationship between mitochondrial and nuclear genetic differentiations.

Table S5 – Outputs of the four-step process following Evanno et al to calculate ΔK [2].

| **# K** | **Reps** | **Mean LnP(K)** | **Stdev LnP(K)** | **Ln'(K)** | **\|Ln''(K)\|** | **Δ K** |
| --- | --- | --- | --- | --- | --- | --- |
| 1 | 5 | -33122,5 | 0,7246 | NA | NA | NA |
| 2 | 5 | -33592,62 | 36,8153 | -470,12 | 447,42 | 12,153096 |
| 3 | 5 | -33615,32 | 126,9436 | -22,7 | 534,02 | 4,20675 |
| 4 | 5 | -34172,04 | 129,0306 | -556,72 | 1026,98 | 7,959196 |
| 5 | 5 | -33701,78 | 81,7719 | 470,26 | 657,74 | 8,043598 |
| 6 | 5 | -33889,26 | 414,23 | -187,48 | 163,18 | 0,393936 |
| 7 | 5 | -33913,56 | 374,8048 | -24,3 | 61,1 | 0,163018 |
| 8 | 5 | -33876,76 | 389,9178 | 36,8 | 36,46 | 0,093507 |
| 9 | 5 | -33803,5 | 416,5669 | 73,26 | NA | NA |

Table S6 - Estimates for the modes and respective 95% confidence interval for mutation-scaled effective population size (θ) and N_e_m in model II, where 4N_e_m=Mj->i*θ for 2010

| *Parameter* | *mode* | *2.50%* | *97.50%* |
| --- | --- | --- | --- |
| *θ* A | 0.067 | 0 | 3.867 |
| *θ* B | 2.067 | 0 | 5.2 |
| *θ* C | 2.467 | 0 | 5.867 |
| *N_e_m* _B 🡪 A_ | 0.325 | 0 | 9.167 |
| *N_e_m* _C 🡪 A_ | 0.318 | 0 | 9 |
| *N_e_m* _A 🡪 B_ | 10.161 | 0 | 9.167 |
| *N_e_m* _C 🡪 B_ | 8.095 | 0 | 9.167 |
| *N_e_m* _A 🡪 C_ | 11.716 | 0 | 9 |
| *N_e_m* _B 🡪 C_ | 9.661 | 0 | 7.667 |

Table S7 - Estimates for the modes and respective 95% confidence interval for mutation-scaled effective population size (θ) and N_e_m in model II, where 4N_e_m=Mj->i*θ for 2011

| *Parameter* | *mode* | *2.50%* | *97.50%* |
| --- | --- | --- | --- |
| *θ* A | 2.6 | 0 | 5.733 |
| *θ* B | 2.067 | 0 | 5.2 |
| *θ* C | 4.6 | 0.4 | 8.533 |
| *N_e_m* _B 🡪 A_ | 14.084 | 0 | 55.423 |
| *N_e_m* _C 🡪 A_ | 12.350 | 0 | 51.6 |
| *N_e_m* _A 🡪 B_ | 11.195 | 0 | 50.267 |
| *N_e_m* _C 🡪 B_ | 8.738 | 0 | 43.325 |
| *N_e_m* _A 🡪 C_ | 21.850 | 0.2 | 76.8 |
| *N_e_m* _B 🡪 C_ | 19.550 | 0.267 | 71.11 |

Table S8 - Estimates for the modes and respective 95% confidence interval for mutation-scaled effective population size (θ) and N_e_m in model II, where 4N_e_m=Mj->i*θ for 2012.

| *Parameter* | *mode* | *2.50%* | *97.50%* |
| --- | --- | --- | --- |
| *θ* A | 0.067 | 0 | 113.33 |
| *θ* B | 0.067 | 0 | 126.67 |
| *θ* C | 4.2 | 0.667 | 145.07 |
| *N_e_m* _B 🡪 A_ | 0.295 | 0 | 28.333 |
| *N_e_m* _C 🡪 A_ | 0.328 | 0 | 31.668 |
| *N_e_m* _A 🡪 B_ | 0.295 | 0 | 48.357 |
| *N_e_m* _C 🡪 B_ | 0.195 | 0 | 29.155 |
| *N_e_m* _A 🡪 C_ | 20.650 | 0.333 | 72.2 |
| *N_e_m* _B 🡪 C_ | 12.250 | 0 | 51.933 |

.

**Supplementary text:**

**1. Amplification and genotyping of microsatellite *loci***

Amplification took place in four PCR multiplexes of four to six loci. Specifically, multiplex A – annealing 55°C (CT77; CT87; CA55; CA58; CT68; AJTR-37), multiplex B - annealing 55°C (CT82; CT76; CT89; CT59; CA80; CT53), multiplex C - annealing 60°C (C01; M23; AJTR-45; AJTR27; I14; O08), multiplex D - annealing 60°C (AJTR-42; B09; B22; N13). All reactions were performed in a total volume of 10 μl and followed the QIAGEN^©^ Multiplex PCR kit’s recommendations. Genotyping was performed on an ABI^©^ 3100 Genetic Analyzer. Alleles were called in GENEMARKER^©^ v. 1.91 (Softgenetics LLC, State College, PA).

**Supplementary References**

Cornuet JM, Luikart G (1996) Description and power analysis of two tests for detecting recent population bottlenecks from allele frequency data. *Genetics* **144**, 2001-2014.

Narum SR (2006) Beyond Bonferroni: less conservative analyses for conservation genetics. *Conservation genetics* **7**, 783-787.
