# Supplementary material for: Does asymmetric gene flow among matrilines maintain the evolutionary potential of the European eel?
Source: Ecol Evol. 2016 Jun 30;6(15):5305–20. doi: 10.1002/ece3.2098 (PMC4984505; doi:10.1002/ece3.2098)
Supplement: Supplementary file 10 [file ECE3-6-5305-s010.docx]

**Supplemental information**

**Appendix 1**

Mitochondrial DNA sequences (ND5)

**Appendix 2**

Microsatellite allelic frequencies

**1. Caption for supplemental figure 1**

Haplotype network, with all shortest trees considered, with explicit mutation steps and frequencies of each haplotype >2. The color code for each matriline is the following: A = Black, B = Yellow and C = Red.

**2. Caption for supplemental figure 2**

Posterior’s marginal likelihood probability distributions of the BEAST runs for each matrilineage. The x-axis represents the posterir while the y-axis represents the density, or the explored parameter space. The effective sample sizes (ESS) of the posterior parameter of each run were as following: A = 219, B = 456, C = 1249. These plots were produced in Tracer (Rambaut A 2014).

**3. Caption for supplemental figure 3**

Graphical display of the simulated confidence areas for each of the respective modes of evolution. Blue dots and respective labels correspond to the markers used in this study. This pattern is common to the infinite allele and stepwise mutation modes of evolution and shows all loci behaving as candidate neutral. The x-axis depicts the expected heterozigosity (He) while the y-axis the F_ST_.

**4. Caption for supplemental figure 4**

Evanno’s *ΔK* calculated has (*ΔK*= mean(|L``(*K*)|)/sd(L(*K*))(Evanno *et al.* 2005). The y-axis represents *ΔK* from K = 2 to K = 9 (x-axis). The modal value of the distribution is the most likely number of clusters. Although peaks were observed in K =2 and K=4 , it is worth mentioning that the Evanno’s method cannot detect K = 1 (Evanno *et al.* 2005).

**5. Caption for supplemental figure 5**

STRUCTURE admixture plots for the modal distributions of K = 2 and K = 4 identified as possible K’s after (Evanno *et al.* 2005). Symmetry across both plots suggests that K = 1 is the most likely number of K.

**6. Caption for supplemental figure 6**

Posterior distributions of migrations rates summed over all the loci for each cohort. The direction of migration is shown with the symbol -> , while the numbers “1”, “2” and “3” correspond to the matrilineages “A”, “B” and “C” respectively.

Bandelt HJ, Forster P, Rohl A (1999) Median-joining networks for inferring intraspecific phylogenies. *Molecular Biology and Evolution* **16**, 37-48.

Evanno G, Regnaut S, Goudet J (2005) Detecting the number of clusters of individuals using the software STRUCTURE: a simulation study. *Molecular Ecology* **14**, 2611-2620.

Le Cren E (1951) The length-weight relationship and seasonal cycle in gonad weight and condition in the perch (Perca fluviatilis). *The Journal of Animal Ecology*, 201-219.

Rambaut A SM, Xie D & Drummond AJ (2014) Tracer v1.6. *Available from* [*http://beast.bio.ed.ac.uk/Tracer*](http://beast.bio.ed.ac.uk/Tracer).
